# Food Sharing With Choice: Influence on Social Evaluation

**DOI:** 10.3389/fpsyg.2020.02070

**Published:** 2020-08-25

**Authors:** Chujun Wang, Jianping Huang, Jiangqun Liao, Xiaoang Wan

**Affiliations:** ^1^Department of Psychology, Tsinghua University, Beijing, China; ^2^Department of Psychology, Soochow University, Suzhou, China

**Keywords:** food sharing, social evaluation, prosociality, choice, eating together

## Abstract

We conducted two studies to investigate the influence of food sharing on people’s social evaluation. In Study 1, the results of an online survey revealed that Chinese adults expected voluntary food sharing to influence the recipient’s social evaluation of the sharer. In Study 2, we ran a laboratory-based experiment in which each participant broke bread with one of two unacquainted individuals. When the participants could choose whom to share food with, they rated the selected person as being more prosocial than the person they did not choose. These results demonstrate the influence of voluntary food sharing with choice on people’s social evaluation of unacquainted individuals, and shed some light on the influence of eating behavior on social perception.

## Introduction

In everyday life, food sharing is very common, especially when people are eating a meal together with family, friends, or colleagues, namely commensality (Fischler, 2011). Food sharing between two adults while eating together often indicates intimacy between them (Miller et al., 1998; Erwin and Burke, 2002). Moreover, eating-together with family and friends can function as an approach to facilitating social bonding and/or engaging in a happy and satisfying life (Julier, 2013; Dunbar, 2017; Masson et al., 2018). Strikingly, even eating together with an unacquainted individual can influence a person’s rating of the food. In Boothby et al.’s (2014) study, participants perceived tasty chocolates to be more enjoyable when each of them was eating together with an unacquainted individual than when eating alone, and perceived awful chocolates to be more unpleasant when eating together than when eating alone.

Boothby et al.’s (2014) demonstrations of the amplification effect of eating-together shed some light on how food sharing may influence people’s food evaluation. However, strictly speaking, eating together with an unacquainted individual in a psychology laboratory may not be considered as food sharing, as food sharing often requires more interactions and communication between individuals (Boothby et al., 2017; Bhargave et al., 2018). Moreover, eating together in a psychology laboratory is usually arranged by the experimenter, whereas natural food sharing in everyday life is either spontaneously initiated by the person having the food (sharer), or requested by the recipient (Birch and Billman, 1986). De Backer et al. (2015) highlighted the differences between eating-together scenes with or without food sharing using examples of restaurant scenes: (1) while eating together, people can have dishes of food placed centrally on the table and divided into portions for each individual; or (2) they can each have their dish of favorite food. In the former case, people are considered to be sharing a meal; whereas in the latter case, they are mainly sharing meal experiences in each other’s presence, though some occasional food sharing also occurs, e.g., one person may invite another person to take a bite of his or her own food.

More importantly, it remains unclear how food sharing influences people’s social evaluation of another person they are sharing food with. Social evaluation refers to a mental process in which people assign positive or negative values to certain social behaviors, associate these behaviors with specific individuals, and show different preference or avoidance toward these individuals (Abdai and Miklósi, 2016). The capability to distinguish prosocial and antisocial partners is crucial for survival (Bonnie and Earley, 2007). Sharing is a typical prosocial behavior (Batson and Powell, 2003; Dunfield, 2014), and food sharing is closely connected to cooperation and trust (Kaplan and Gurven, 2005; Woolley and Fishbach, 2019). Therefore, it seems reasonable to expect a recipient to generate social evaluation in favor of a food sharer.

It should also be noted that humans spontaneously make inferences regarding an individual’s traits without intention, attention, or effort (Krull and Dill, 1996; Uleman et al., 2008). Trait concepts and stereotypes can be automatically activated when relevant behaviors are perceived (Bargh et al., 1996). One of such traits is a person’s prosociality, which refers to positive behaviors, intentions, emotions, and attitudes directed toward people other than the self (Knafo-Noam et al., 2015). For example, when people see an unacquainted individual sharing his or her food, they may quickly make inferences about his or her prosociality. Therefore, it seems reasonable to expect that even one-time food sharing between unacquainted individuals may influence the recipient’s social evaluation of the sharer’s prosociality.

Moreover, the perceived autonomy of the recipient may also play a key role in the formation of such prosociality evaluation. Autonomy refers to the sense of behaving freely and choicefully (Kasser and Ryan, 1999). People have a certain need for autonomy, and like making choices for the self (Deci and Ryan, 2000, 2012). Therefore, the perceived autonomy of a food sharer may be important for the recipient’s social evaluation, as previous research has demonstrated that recipients of prosocial acts experience more gratitude toward helpers with autonomous motivations than those with introjected motivations (Weinstein et al., 2010). The autonomy of a food recipient is also important, as previous research has shown that a recipient of unneeded and unsolicited helping in a task might show lower task-based self-esteem and become more depressed (Schneider et al., 1996). The recipient’s need for autonomy may be fulfilled by choosing for themselves (Van Doesum et al., 2013), such as choosing whose invitation of food sharing they would accept.

Therefore, we conducted two studies to investigate the influence of food sharing on people’s prosociality evaluation of unacquainted individuals, and focused on the recipient’s evaluation of an unacquainted individual whom they chose to accept food sharing. We are interested in two research questions. First, do people implicitly associate the act of food sharing with sharing partners’ prosociality? Second, does the act of food sharing have to be voluntary to influence people’s evaluation of a sharer’s prosociality? These questions were addressed in two studies, respectively. To preview, in Study 1, we surveyed young adults from mainland China about their beliefs regarding food sharing, which is an integral part of Chinese food culture and is valued in the preparation, serving, and consumption of food (Ma, 2015). In Study 2, we ran a laboratory-based food-sharing experiment to examine the effect of food sharing on Chinese participants’ social evaluation of unacquainted individuals.

## Study 1

In this study, we surveyed young adults from mainland China about their beliefs regarding food sharing. Most importantly, we probed the association between food sharing and the perception of the sharing partner’s prosociality. In order to clearly differentiate food sharing and eating together (De Backer et al., 2015), we also examined the extent to which different eating-together scenarios matched participants’ definition of food sharing.

### Materials and Methods

#### Participants

Two hundred Chinese adults (mean age = 20.0 ± 1.8 years, ranging from 18 to 26 years; 100 males and 100 females) were recruited from three major Chinese cities (Beijing, Chongqing, and Suzhou) to take part in this study. The present and the following studies were approved by the ethics committee of the Psychology Department of Tsinghua University, and performed in accordance with the ethical standards laid down in the Declaration of Helsinki. All participants gave informed consents electronically before the experiment started. They either volunteered to take part in the present study or received 10 Chinese Yuan for their time and participation. We used the G^∗^Power software to estimate the sample size, and the results revealed that a sample of 200 participants can detect the effects with $\eta_{\text{p}}^{2}$ ≥ 0.09 (statistical power = 0.95).

#### Design and Procedure

The participants were asked to complete a survey online on the website of www.wjx.cn. To disguise the real purpose of the study, we first asked the participants to rate the extent to which different types of food were appropriate for sharing, and the extent to which they would be willing to share food with a random person with certain characteristics such as age and sex etc. After that, we asked the first set of questions that were relevant to the real purpose of the present study. In order to probe the possible association between food sharing and prosociality evaluation, we asked the participants to rate the extent to which they would be willing to share food with a friendly, unfriendly, generous, or ungenerous individual, and the extent to which they expected this person to be willing to share food with them (1 = not willing at all, and 7 = very willing).

Second, we created brief descriptions of four eating scenarios to test how each scenario would influence the participants’ social evaluation: (1) “you and another person are eating food at the same time and place, but each of you is eating your own food,” (2) “you and another person are arranged by a third party to split and eat food together,” (3) “another person invites you to split and eat food together with him or her,” and (4) “you invite another person to split and eat food together with you.” Thus, the eating partner is described as a parallel eater, an involuntary sharer, a voluntary sharer, or a sharing recipient, respectively. Each participant was asked to rate how much eating together would influence his or her evaluation of the eating partner (1 = no influence at all, and 7 = great influence). Here it should be noted that the “influence” can refer to positive or negative influence, and we chose not to be specific about positive influence to avoid the possible halo effect of the term “sharing.” Similarly, we also asked the participants to rate the influence of eating together on the partner’s evaluation of them if the participant was the parallel eater, involuntary sharer, voluntary sharer, or sharing recipient.

Third, the participants were asked to indicate the extent to which each eating scenario matched their definition of food sharing (1 = does not match at all, and 7 = completely matches). The scenarios being rated were “you eat alone” and the four eating-together scenarios mentioned above, namely eating in parallel with another person, splitting and eating with another person arranged by a third party, being invited to split and eat with another person, as well as inviting another person to split and eat together.

### Results

The mean willingness-to-share scores are summarized in Table 1. In order to probe the associations between food sharing and prosocial evaluation, we performed two of one-way repeated-measure ANOVAs on the participants’ willingness to share food with friendly/unfriendly and generous/ungenerous individuals. The results revealed that the participants were more willing to share food with a friendly person than with an unfriendly person, and they were more willing to share food with a generous person than with an ungenerous person, both *F*s > 562.78, *p*s < 0.001, $\eta_{\text{p}}^{2}$ > 0.73. We also performed analogous analyses on the participants’ estimation of other individuals’ willingness to share food. The results revealed that the participants expected greater willingness to share food from a friendly person than from an unfriendly person, and greater willingness from a generous person than from an ungenerous person, both *F*s > 605.57, *p*s < 0.001, $\eta_{\text{p}}^{2}$ > 0.74.

**TABLE 1:** Mean ratings of willingness to share (with SD in parentheses) in Study 1.

	**Willingness to share with this person**	**This person’s willingness to share**
A friendly person	6.3 (1.0)	6.0 (1.1)
An unfriendly person	3.0 (1.7)	2.9 (1.6)
A generous person	6.3 (1.0)	6.0 (1.1)
An ungenerous person	2.2 (1.6)	2.0 (1.4)

The mean ratings of the influence of eating together on social evaluation are summarized in Table 2. In order to test the effects of eating together on social evaluation, we performed a 4 (Evaluatee: parallel eater, involuntary sharer, voluntary sharer, or sharing recipient) × 2 (Evaluator: self or another person) repeated-measure ANOVA on these scores. The results revealed a significant main effect of Evaluatee, *F*(3,597) = 31.44, *p* < 0.001, $\eta_{\text{p}}^{2}$ = 0.14; whereas neither the main effect of Evaluator nor the interaction term reached the significance level, both *F*s < 3.28, *p*s > 0.07. Pairwise comparisons with Bonferroni correction revealed that the participants expected voluntary food sharing to exert the greatest influence on people’s social evaluation of the eating partner (*M* = 4.6, *SD* = 1.6), all *t*s > 5.56, *p*s < 0.001, Cohen’s *d*s > 0.37. Moreover, both involuntary food sharing (*M* = 3.9, *SD* = 1.7) and receiving sharing (*M* = 4.1, *SD* = 1.6) were expected to exert greater influence than eating in parallel (*M* = 3.5, *SD* = 1.6), both *t*s > 3.74, *p*s < 0.01, Cohen’s *d*s > 0.22, whereas the difference between involuntary sharing and receiving sharing was not significant, *t*(199) = 1.30, *p* > 0.99.

**TABLE 2:** Mean ratings of the influence of eating-together on social evaluation (with SD in parentheses) in Study 1.

**Evaluatee**	**Evaluator**	
	**Self**	**Another person**
Parallel eater	3.4 (1.8)	3.5 (1.8)
Involuntary sharer	3.9 (1.8)	3.9 (1.7)
Voluntary sharer	4.6 (1.7)	4.7 (1.7)
Sharing recipient	4.0 (1.7)	4.1 (1.8)

In order to probe the participants’ definition of food sharing, we then conducted a one-way repeated-measure ANOVA on the definition-matching scores of eating scenarios. The results revealed a significant main effect of Scenario, *F*(4,796) = 88.05, *p* < 0.001, $\eta_{\text{p}}^{2}$ = 0.31. Planned pairwise comparisons with Bonferroni correction revealed that inviting another person to share food with oneself was the most consistent with our participants’ definition of food sharing (*M* = 5.5, *SD* = 1.6), all *t*s > 2.94, *p*s < 0.037, Cohen’s *d*s > 0.26. They also considered the scenario of being invited by someone else to share (*M* = 5.2, *SD* = 1.7) to be more consistent with their definition of food sharing than the other three scenarios, all *t*s > 8.65, *p*s < 0.001, Cohen’s *d*s > 0.61. Moreover, the scenario of eating in parallel (*M* = 3.4, *SD* = 1.9) matched their definition of food sharing more than the scenario of eating alone (*M* = 3.1, *SD* = 2.1), *t*(199) = 3.26, *p* = 0.013, Cohen’s *d* = 0.20, whereas neither of these two scenarios significantly differed from the scenario of sharing arranged by a third party (*M* = 3.2, *SD* = 1.7), both *t*s < 1.06, *p*s > 0.99.

### Discussion

In summary, two major findings emerged from this study. First, the participants indicated that they preferred to share food with a generous or friendly person rather than an ungenerous or unfriendly person. They also expected voluntary food sharing to influence the recipient’s social evaluation of the sharer, even though we did not specifically ask them to indicate whether the influence would be positive or negative. Collectively, these results demonstrate the associations between food sharing and people’s perception of the sharing partners’ prosociality, which is in line with our research hypothesis. Considering that sharing is a typical prosocial behavior (Batson and Powell, 2003; Dunfield, 2014), it is very likely that the recipient of food sharing generates a positive bias toward the food sharer (Abdai and Miklósi, 2016).

Second, our participants defined food sharing as being spontaneously initiated by the sharer or recipient (see also Birch and Billman, 1986), whereas eating in parallel with another person and being assigned by a third party to share food with another person were hardly defined as food sharing. It should be noted that these two latter eating scenarios also involve eating together with another person. Therefore, these results demonstrate the important difference between food sharing and eating together (De Backer et al., 2015), and emphasize the key role of both sharer’s and recipient’s autonomy in the act of food sharing.

Based on the results of Study 1, we created a laboratory-based scenario in Study 2 and had each participant share food with one of two experimental confederates. We also had the participants and two experimental confederates eat together after food sharing, to control for the possible influence of eating together (Boothby et al., 2014). Then we compared the participants’ social evaluation of the two unacquainted individuals.

## Study 2

In this study, we ran a laboratory-based experiment to examine whether food sharing can influence the recipient’s social evaluation of the sharer. In order to simulate the spontaneous act of food sharing as naturally as possible in a laboratory-based scenario, we developed a novel experimental paradigm in which we invited the participants to taste a new jam product and had each of them split bread with another person. Importantly, we created two experimental conditions of voluntary food sharing differing in whether the participants were able to experience autonomy by choosing their sharing partners or not. Based on the results of Study 1, we also created a condition of involuntary food sharing in which food sharing was arranged by an experimenter. In order to control for the influence of demand characteristics (Weber and Cook, 1972), we indirectly measured the participants’ social evaluation of other people by asking them to predict the helping and donation intentions toward a third party (Prochazka and Vaculik, 2011).

### Materials and Methods

#### Participants

Ninety young Chinese adults (mean age = 20.6 ± 2.0 years, ranging from 18 to 25 years) were recruited from a major research university in Beijing to take part in this study. None of them took part in Study 1 and each of them received 30 Chinese yuan (CNY) for participation. Considering that Boothby et al. (2014) only tested female participants in their eating-together experiment, we used a similar experimental design and only tested female participants in this study.

All participants were randomly and evenly divided into three groups (with 30 participants in each group) and completed the experiment under different conditions. We used the G^∗^Power software to estimate the sample size, and the results revealed that a sample of 30 participants in each group can detect the effects with $\eta_{\text{p}}^{2}$ ≥ 0.30 (statistical power = 0.90).

#### Design and Procedure

This study was conducted in a conference room in which we placed a conference desk and a few conference chairs for the participants to complete surveys, as well as a round table and three foldable chairs for eating. The food stimuli were a slice of white bread (50 g) and a tiny box of apricot jam (14.2 g). Even though bread and jam are not typical Chinese foods, they are quite common in breakfast for the population of Chinese young adults from which we recruited our participants. In order to control for the cross-sex differences, all experimenters and experimental confederates were females. We trained a total of seven research assistants to be experimental confederates. Two research assistants were randomly chosen for each participant, and the experimenter made sure that neither of them had previously met the participant.

Each participant was invited to take part in a jam tasting individually. When she arrived at the conference room, two experimental confederates were already present, posing as two other participants. The experimenter informed them that the jam was to be spread on bread and eaten all together. Then the experimenter cut a slice of bread in two halves, and informed them that two of them would share one half-slice of bread, whereas the third person would eat one half of the other half-slice of bread and return the rest. The experimenter then placed the two half-slices of bread on the table.

Under the condition of voluntary sharing without choice, one of the confederates (randomly determined prior to the experiment) invited the participant to share one half-slice of bread with her. Under the condition of voluntary sharing with choice, both confederates asked the participant to share a half-slice of bread with them at approximately the same time, so the participant had a chance to make a decision about whom to share with. Under the condition of involuntary sharing, the experimenter delivered one half-slice of bread to one of the confederates and asked the other confederate and the participant to split the other half-slice of bread. When splitting the bread, two people each held one side of the bread and tore it apart into two comparable pieces. Each participant also shared one package of jam with the same confederate she split bread with, and used her own knife to spread the jam on the bread. Then the participant and two confederates were instructed to eat the bread and jam all together, so the influence of eating-together was well controlled for.

After that, the participants were asked to complete some rating tasks on 7-point scales. In order to disguise the real purpose of this study, we first asked the participants to rate the taste of the jam and bread, and then asked them to guess the other two people’s answers to the same questions. Second, each participant was asked to rate the willingness of everyone (including herself and the other two people) to help the experimenter enter survey data into the computer. Third, the participants were asked to rate everyone’s likelihood of donating some of their monetary compensation for taking part in this study (30 CNY) to the United Nations International Children’s Emergency Fund (UNICEF).

### Results

**FIGURE 1:**
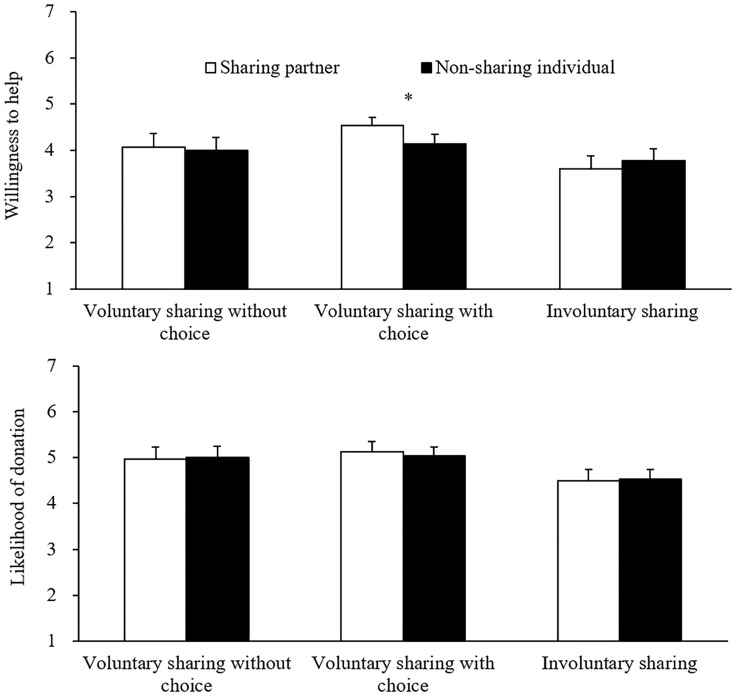
Mean ratings of the sharing partner’s and non-sharing individual’s willingness to help to the experimenter and likelihood of donating to the UNICEF in Study 2. The error bars show the standard errors of the means, and * denotes *p* < 0.05.

The participants’ mean ratings of the other two people’s willingness to help the experimenter and likelihood of donating to the UNICEF are shown in Figure 1. We performed 2 (Evaluatee: sharing partner or non-sharing individual) × 3 (Sharing: voluntary sharing without choice, voluntary sharing with choice, or involuntary sharing) mixed-design ANOVAs on these data, with Evaluatee being a within-participants factor and Sharing being a between-participants factor. The results revealed a significant interaction term on the willingness-to-help ratings, *F*(2,87) = 3.35, *p* = 0.040, $\eta_{\text{p}}^{2}$ = 0.07, whereas neither of the two main effects was significant, both *F*s < 1.84, *p*s > 0.16. By contrast, none of the main or interaction effects was significant on the likelihood of donation scores, all *F*s < 1.91, *p*s > 0.15.

In order to interpret this significant interaction term on the willingness-to-help ratings, we performed one-way repeated-measure ANOVA for each type of sharing, with Evaluatee (sharing partner or non-sharing individual) as the independent variable. As for voluntary sharing with choice, the participants rated the sharing partner (*M* = 4.5, *SD* = 0.9) as being more willing to help the experimenter than the non-sharing individual (*M* = 4.1, *SD* = 1.2), *F*(1,29) = 6.57, *p* = 0.016, $\eta_{\text{p}}^{2}$ = 0.19, indicative of a significant influence of food sharing on prosociality evaluation. By contrast, no such effect was significant for voluntary food sharing without choice, *F*(1,29) = 0.15, *p* = 0.70, or involuntary food sharing, *F*(1,29) = 1.50, *p* = 0.23.

### Discussion

The results of this study revealed the influence of voluntary food sharing with choice on the recipient’s evaluation of the sharer. Importantly, voluntary food sharing with choice resulted in a more positive evaluation of the sharer’s willingness-to-help for a third party than a non-sharing parallel eater. This effect was not significant on the participants’ ratings of other people’s likelihood of donation to charity, and this discrepancy between two measures may be attributed to the difference between helping and donation (Lee et al., 1999). That is, helping the experimenter to input data only requires time and effort, whereas donating to charity has a monetary cost. The mentioning of money might make people focus more on personal goals or resources, which could interfere with their prosocial intentions (Vohs et al., 2006; Chatterjee et al., 2013). Moreover, it should be noted that all of our participants were recruited to take part in this study in exchange for monetary compensation, and our questions involved donating a person’s payment for this study to charity. Therefore, the willingness-to-help measure may be a more sensitive measure than the likelihood-of-donation measure for the evaluation of other people’s prosociality in this study.

By contrast, the recipient’s evaluation of the sharing and non-sharing individuals did not differ when food sharing was arranged by the experimenter, or when food sharing was initiated by a sharer but the participants had no choice about whom they shared food with. Based on the results of Study 1, it is very likely that our participants under the involuntary food sharing condition did not consider assigned eating-together as food sharing, so they might consider both of the other two individuals as parallel eaters. As for the condition of voluntary food sharing without choice, only one experimental confederate invited each participant to break food with her, and the participants did not show any significant differences in their social evaluation of this invitee/sharer and the parallel eater. This result suggests that the perceived autonomy of the sharer is not sufficient to elicit a more positive evaluation of an unacquainted individual’s prosociality. The recipient’s autonomy in one-time food sharing is also important for the formation of prosociality evaluation, suggesting that the influence of one-time food sharing on people’s social evaluation may be subtle and subject to situational factors (Amici, 2015).

## General Discussion

We conducted two studies to examine the influence of food sharing on people’s social evaluation of unacquainted individuals. The results of Study 1 suggest that young Chinese adults do associate food sharing with the evaluation of a sharer’s prosociality. The results of Study 2 suggest that a recipient of one-time voluntary food sharing with choice would have a more positive social evaluation of the sharer’s prosocial intentions toward other people. Collectively, these results suggest that food sharing is closely connected to cooperation and trust (Kaplan and Gurven, 2005; Woolley and Fishbach, 2019). These results are also in line with the literature that people can make inferences about other people’s traits based on a very limited amount of information (Winter and Uleman, 1984; Oosterhof and Todorov, 2008).

By contrast, no significant effects were observed for voluntary food sharing without choice in Study 2. This result is consistent with the notion that people do have certain need for autonomy (Deci and Ryan, 2000, 2012), and perceived autonomy is important for social interaction (Deci et al., 2006), especially for the recipients of prosocial acts (Schneider et al., 1996; Van Doesum et al., 2013). In Study 2, the participants did not need to share food with anyone if one confederate was simply asking the other confederate to share with her. When a participant was invited by only one confederate for food sharing, it is possible that she did not have a feeling of choice (Savani et al., 2010; Kouchaki et al., 2018), and even felt that they were “pushed” or compelled to share food with the only person who asked them. Therefore, in the condition of voluntary food sharing with choice, the participants’ need for autonomy was fulfilled when they chose whose invitation of food sharing they would accept, whereas the participants under the condition of voluntary food sharing without choice did not have this chance.

Considering that both confederates were unacquainted individuals for the participants, it is possible that the participants did not have any preference for either of these two people at the beginning, but making a choice resulted in a more favorable evaluation of the chosen person (Brehm, 1956). Making a choice can create an association between oneself and the chosen option (Gawronski et al., 2007), and the motive to stay self-consistent (Beaman et al., 1983) can also result in acts congruent with the previous choice, such as that predicting the chosen individual to be more prosocial as in our Study 2.

As with any study, there are certain limitations to the present study. First, considering that food sharing is shaped by culture (Ringen et al., 2019), caution is called for in trying to generalize our findings in Studies 1 and 2 with Chinese adults to other populations. Second, we only recruited female participants in our Study 2, but it will be important to test male participants in future research. Third, we only conducted a laboratory-based experiment in Study 2, but it is important to test with more realistic settings to enhance the ecological validity of the results in future research. Fourth, it is also interesting to examine the moderating effect of individual differences in eating-related factors, such as eating disorders, obesity, or food addiction (Innamorati et al., 2014b; Imperatori et al., 2017). Individual differences in relationship-related factors may be also considered as covariates in future research. For example, individuals with high rejection sensitivity may react defensively to interpersonal relationships (Downey and Feldman, 1996; Innamorati et al., 2014a), and individuals with affect lability or cognitive vulnerabilities may show abnormally or negatively affective states while eating with other people (Balsamo et al., 2013; Contardi et al., 2018).

In conclusion, the results of this study provide empirical evidence regarding the difference between food sharing and eating together (De Backer et al., 2015), and demonstrate the influence of voluntary food sharing with choice on people’s social evaluation of other individuals. Specifically, choosing an unacquainted individual to break bread with oneself is sufficient to lead to a more positive evaluation of this selected person. The decision-making literature has demonstrated that choosing an object shapes people’s preference (Brehm, 1956; Kitayama et al., 2013; Voigt et al., 2017), and the present study has extended this finding to the domain of social evaluation of people. The choosing-one-to-share-food-with paradigm we developed may also have implications in the implicit measure of person evaluation, or function as another foot-in-the-door strategy to obtain a more positive outcome in negotiation or persuasion.

## Data Availability Statement

The raw data supporting the conclusions of this article will be made available by the authors, without undue reservation, to any qualified researcher.

## Ethics Statement

The studies involving human participants were reviewed and approved by Ethics Committee of the Psychology Department of Tsinghua University. The patients/participants provided their written informed consent to participate in this study.

## Author Contributions

XW and JL co-developed the idea for the study and collaboratively designed the study. CW and JH collected and analyzed the data, and conducted the interpretation of the data. CW and XW drafted the manuscript. JH provided critical revisions. All authors have approved the final version of the manuscript.

## Conflict of Interest

The authors declare that the research was conducted in the absence of any commercial or financial relationships that could be construed as a potential conflict of interest.
